# Antifungal Susceptibility of Clinical *Meyerozyma guillermondii* Isolates Obtained Between 1994 and 2014: Original Research and Comparison with Published Data

**DOI:** 10.3390/pathogens15020235

**Published:** 2026-02-20

**Authors:** Aleksandra Górzyńska, Daria Konarska, Agnieszka Korzeniowska-Kowal, Anna Wzorek, Bartosz Pencakowski, Urszula Nawrot

**Affiliations:** 1 Department of Pharmaceutical Microbiology and Parasitology, Wrocław Medical University, Borowska 211a, 50-556 Wrocław, Polandurszula.nawrot@umw.edu.pl (U.N.); 2 Department of Immunology of Infectious Diseases, Hirszfeld Institute of Immunology and Experimental Therapy, Polish Academy of Sciences, St. Weigla 12, 53-114 Wrocław, Poland; agnieszka.korzeniowska-kowal@hirszfeld.pl (A.K.-K.); anna.wzorek@hirszfeld.pl (A.W.); 3 Department of Pharmaceutical Biology and Biotechnology, Wrocław Medical University, 50-556 Wrocław, Poland; bartosz.pencakowski@umw.edu.pl

**Keywords:** *Meyerozyma guillermondii*, azoles, echinocandins, amphotericin B, flucytosine, manogepix

## Abstract

(1) Background: *Meyerozyma guilliermondii* is a yeast species widely distributed in the natural environment and one of the rare emerging pathogens capable of causing difficult to treat, severe infections. The species’ susceptibility profile is not fully defined; however, the species could be more prone to develop resistance than other *Candida* species. The objective of this research was to determine the susceptibility of a local collection of *Meyerozyma guilliermondii* clinical isolates to classical antifungal drugs as well as a new one—manogepix. (2) Methods: The study included 20 clinical isolates identified using the MALDI–TOF method followed with sequencing of ITS1-2 region of DNA. Overall, the susceptibility to 12 antifungal drugs was tested. Nine drugs (amphotericin B, flucytosine, fluconazole, itraconazole, posaconazole, voriconazole, anidulafungin, caspofungin, and micafungin) were assessed using the MICRONAUT–AT test. The susceptibility to the new drug, manogepix, as well as isavuconazole, clotrimazole and anidulafungin, was determined using the microdilution method recommended by EUCAST. Additionally, anidulafungin and voriconazole MIC was also examined with commercial gradient tests. (3) Results: Overall, the isolates showed low MIC values for amphotericin B (0.125 to 1 mg/L) and for flucytosine (≤0.06 to 32 mg/L), with the exception of one isolate with a high MIC value. The MIC ranges for azoles were 2–64 mg/L (fluconazole), 0.008–0.5 mg/L (voriconazole), ≤0.03–≥4 mg/L (itraconazole) and 0.008–0.5 mg/L (posaconazole). One isolate showed non-WT phenotype to all tested azoles. For anidulafungin, the MIC values ranged from ≤0.06 to 0.25 mg/L; however, in the reference method, higher values were observed, but they did not exceed 2 mg/L (ECOFF value). For manogepix, the MIC values ranged from 0.002 to 0.125 mg/L. Finally, the comparison of the obtained and published susceptibility data was conducted. (4) Conclusions: The data obtained in this study are consistent with reports by other authors and indicate that resistance to azoles or 5-fluorocytosine among clinical isolates of *Meyerozyma guilliermondii* should be considered. The low MIC values of manogepix suggest the potentially good efficacy of this drug against *Meyerozyma guilliermondii* species.

## 1. Introduction

Fungal infections have become a growing concern, particularly among immunocompromised individuals, such as cancer patients, and, mainly, those with hematological malignancies and a bone marrow transplant, those in intensive care units, and surgical patients or HIV-positive patients. While some *Candida* species, such as *Candida albicans*, *Nakaseomyces glabratus* (formerly *Candida glabrata*) or *Candida parapsilosis*, are well-recognized pathogens in clinical mycology, the emergence of so-called rare yeast species, such as *Meyerozyma guilliermondii* (formerly *Candida guilliermondii*), has started to gain attention [1]. This species is widespread in nature, being found in soil and water, and can also colonize the human skin. Moreover, this species has been increasingly isolated from clinical specimens, indicating its ability to cause human infections [2]. *Meyerozyma guilliermondii* has been reported as increasing in frequency in recent years, posing risks especially to immunosuppressed patients. This population, particularly those undergoing chemotherapy, organ transplantation or long-term corticosteroid therapy, is often affected by opportunistic fungal infections. *Meyerozyma guilliermondii* has been isolated from severe infections such as fungemia, brain abscesses and urinary tract infections. It is the cause of approximately 2–3% of candidemia cases; however, in some centres, this proportion may be even higher [2,3].

The clinical significance of rare fungal pathogens cannot be overlooked, as their diagnosis and treatment present unique challenges. For instance, *Candidozyma auris*, a fungal pathogen that exhibits resistance to well-known antifungals, including azoles, amphotericin B and echinocandins, has become a globally emerging pathogen [4]. The risks posed by climate change, the migration of people and goods, and the overuse of antifungals and fungicides may create favourable conditions for the spread of other rare yeast species and spur on the development of new infection outbreaks or even epidemics [5]. Therefore, it is extremely important to learn about susceptibility of rare yeasts to antifungal drugs, including *Meyerozyma guilliermondii,* and to continue efforts to develop new antifungals.

Manogepix, the active form of a prodrug fosmanogepix, is a novel antifungal currently in the third stage of clinical trials. Unlike conventional antifungals, manogepix exhibits a unique mechanism of action by targeting the fungal enzyme Gwt1, which is critical for glycosylphosphatidylinositol (GPI) anchor biosynthesis. Furthermore, this inhibition results in pleiotropic effects on the fungal cell, such as the impairment of cell wall mannoprotein localization, compromised cell wall integrity, and an impaired germ tube formation, leading to the inhibition of fungal growth. This innovative approach not only circumvents common resistance mechanisms but also demonstrates a broad spectrum of activity against a wide range of fungal pathogens: yeasts (including *Candida auris*) and moulds (even those considered panfungal resistant, e.g., *Fusarium* spp.) [6,7]. Given its promising efficacy, it is crucial to evaluate the activity of manogepix against local fungal isolates to explore its therapeutic potential further. Testing its effectiveness on these isolates may reveal novel therapeutic options, particularly for addressing emerging resistance patterns.

The drug susceptibility profile of *Meyerozyma guilliermondii* is not fully described, but it is characterized by naturally higher MIC values for echinocandins, and it has also been reported to show resistance to azoles and 5-flucytosine. Overall, it is considered to be a species more prone to developing resistance and even multidrug resistance.

It is important to expand on the existing knowledge of the inherent susceptibility and resistance patterns of this fungal species, as this may be crucial for the selection of effective therapy and infection prevention. Recently, several papers have been published worldwide indicating the increased occurrence of this pathogen in clinical materials [8,9]. Unfortunately, the distribution and susceptibility of *Meyerozyma guilliermondii* in Poland are poorly described. The species is isolated relatively rarely and only individual strains included as a part of larger epidemiological analyses have been published [10]. Therefore, a knowledge gap exists regarding the susceptibility profile of local *Meyerozyma guilliermondii* isolates. The present study examines the collection of clinical isolates of *M. guilliermondii* in order to assess their susceptibility to commonly used and novel antifungals such as manogepix or isavuconazole. The article aimed to present the analysis of the obtained and published data in the form of a mini-review.

## 2. Materials and Methods

### 2.1. Tested Fungi

The study included 20 clinical isolates of the *Meyerozyma guilliermondii* species complex that were part of the collection of the Department of Pharmaceutical Microbiology and Parasitology at the Wroclaw Medical University. All the isolates were obtained from patients of University Clinical Hospital in Wroclaw between 1994 and 2014. Unfortunately, after 2014, our access to the strains was limited and we did not obtain any isolate of this species. The isolates studied were acquired from blood (4), cerebrospinal fluid (1), pericardial fluid (1), wound (1), bronchoalveolar lavage fluid (1), throat swab (3), nasal swab (1), sputum (1), urine (3), foreskin swab (2) and feces (2), with each strain obtained from a different patient. The origins of the particular isolates tested are shown in Supplementary Table S1. All the isolates were initially identified as *Candida guilliermondii*, using phenotypic metabolic tests, and, in this research, the identification was confirmed with MALDI-TOF and ITS1-2 sequencing.

As a reference, three isolates were used: *Meyerozyma guilliermondii* IHEM 19923 (Belgian Coordinated Collections of Microorganisms/Fungi Collection: Human and Animal Health), *Pichia kudriavzevii/Issatchenkia orientalis* ATCC 6258 (American Type Culture Collection) and *Candia parapsilosis* ATCC 22019.

All the isolates were stored frozen at −75 °C, in TSB medium with 40% glycerol. Following defrosting, the isolates were cultured in Sabouraud Dextrose LAB-AGAR™ (SAB) medium (BioMaxima, Poland) and incubated at a temperature of 37 °C for 24 to 48 h.

### 2.2. Methods

#### 2.2.1. MALDI-TOF Performance

The exact procedure of MALDI-TOF identification performance was previously described in the article by Górzyńska et al. [11]. The ready plate was placed in a MALDI Biotyper Sirius mass spectrometer (Bruker Daltonics, Bremen, Germany; laser frequency: 200 Hz) and the mass spectra of the extracted ribosomal proteins were obtained using flexControl, Version 3.4, software. The spectra were calibrated using *E. coli* DH5-alpha standard (Bruker). MBT Compass 4.1 and MALDI Biotyper Compass Explorer 4.1 software were used to identify the mass spectra, as well as the MBT Compass Library Revision H (2022) database (Bruker Daltonics GmbH & Co. KG), containing 3893 species. The identification results were classified according to the manufacturer’s instructions depending on the score:

- 2.300–3.000: Highly probable identification of the species;
- 2.000–2.299: Genus identification; probable identification of the species;
- 1.700–1.999: Probable genus identification;
−1.699: Invalid score; no identification.

#### 2.2.2. Molecular Identification

DNA from all the isolates was extracted according to procedure described by Brillowska-Dąbrowska et al. [12]. Briefly, the DNA was obtained from 24 h colonies grown on Sabouraud medium and extracted via the 10 min incubation of the sample in 100 µL of extraction buffer (60 mM sodium bicarbonate (NaHCO3), 250 mM potassium chloride (KCl) and 50 mM Tris, pH 9.5) at 95 °C and the subsequent addition of 100 µL of 2% bovine serum albumin. After vertexing, this DNA-containing solution was frozen at −20 °C. After defrosting, the DNA was used for the amplification of the ITS regions (ITS1, 5.8S rRNA gene and ITS2), which were amplified from all the investigated isolates using the primers ITS1 (5′-TCCGTAGGTGAACCTGCGG-3′) and ITS4 (5′-TCCTCCGCTTATTGATATGC-3′) [13].

The PCR amplification was performed in 20 μL. The reaction mix was composed of the template DNA, the primers and the PCR mix (EURx, Gdansk, Poland), according to the manufacturer’s instructions. The reactions were carried out in a T-100 thermal cycler (BIO-RAD Laboratories, Hercules, CA, USA) with 2 min of initial denaturation at 98 °C, followed by 35 cycles, involving denaturation (98 °C/15 s) annealing (62 °C/30 s), elongation (72 °C/1 min.) and a final extension at 72 °C for 2 min.

The amplification products were visualized by gel electrophoresis in 2% agarose gel in TBE buffer. The mass marker was 50 bp DNA Ladder (A&A Biotechnology, Gdańsk, Poland). PCR products were purified with a PCR Mini Kit (Syngen Biotech Wroclaw, Poland) from the post-reaction mix. Cycle sequencing reactions were carried out with a BrilliantDye TM Terminator Cycle Sequencing Kit (NimaGen B.V., Nijmegen, The Netherlands) in a twofold dilution of the reaction premix protocol in 10 μL, according to the manufacturer’s instructions. Then, two sequencing reactions were conducted with each primer. The sequencing products were precipitated with ethanol and dissolved in TSR (Life Technologies, Carlsbad, CA, USA).

Capillary Electrophoresis (CE) was conducted on an Applied Biosystems™ 310 Genetic Analyzer (Thermo Fisher Scientific, Waltham, MA, USA). CE was performed at 50 °C, in a 50 cm long capillary filled with POP-7. The separation time ranged from 90 to 150 min and the run voltage was 6 kV with 60 s of 2 kV sample injection. For the base calling, Sequence Scanner Software v2.0 was utilized, and the peak quality was checked with the Sanger Quality Check App. The obtained sequences were identified by comparing them with the NCBI sequence database using the BLAST programme and a match identity of 97% or higher was considered a reliable identification.

#### 2.2.3. Sequence Alignment and Phylogenetic Analysis

In total, 17 ITS sequences from the studied isolates were compiled into a dataset along with 24 sequences retrieved from GenBank. Sequence alignment was performed using the ClustalW algorithm implemented in MEGA 12 [14], which was also utilized for phylogenetic estimation via the Maximum Likelihood method. The alignment was then processed using Gblocks v.0.91b [15] to remove poorly aligned positions and divergent regions with the least stringent parameters. Based on Bayesian Information Criterion (BIC) values, a T92+G+I substitution model was selected [16].

#### 2.2.4. MICRONAUT-AM Test

Antifungal susceptibility testing was performed using MICRONAUT-AM (MERLIN Diagnostic GmbH, Germany) kits, which included pre-prepared 96-well microtiter plates containing a concentration gradient of nine lyophilized antifungal agents: amphotericin B (AmB), flucytosine (FC), fluconazole (FLU), voriconazole (VOR), posaconazole (POS), itraconazole (ITR), anidulafungin (AND), micafungin (MIF) and caspofungin (CAS). The test setup also provided 11.5 mL tubes of colourless RPMI 1640 liquid medium and two indicators: an AST-indicator and methylene blue. The plates were sealed and incubated at 35 °C for 24 h.

According to the manufacturer’s instructions, the results were read visually, where blue indicated the absence of multiplication of the strain, pink indicated fungal growth and no colour indicated intensive fungal growth. The value of the minimum inhibitory concentration (MIC) was set as the lowest concentration of the antimycotic tested (in mg/L) at which the growth of the strain was inhibited (blue). In the case of inconclusive results, the MIC was taken as the concentration at which the blue colour was still observed.

#### 2.2.5. Microdilution Reference Method According to EUCAST

The microdilution method according to EUCAST guidelines (the European Committee on Antimicrobial Susceptibility Testing) was applied to test the susceptibility of isolates to four antifungals, including three not included in MICRONAUT-AM panels: manogepix (MGX) (Sigma-Aldrich), isavuconazole (ISA) (Sigma-Aldrich) and clotrimazole (CLTZ) (Hasco-Lek, Wroclaw, Poland), and one (anidulafungin (AND) (Sigma-Aldrich)), tested as a comparison [17].

For this, sterile 96-well microplates were prepared with serial dilutions of the antifungal agent. Each well contained 100 µL of antifungal in a 2x RPMI-1640 medium. The tested range of dilutions was different for each drug: for MGX, the gradient was 0.5 mg/L–0.001 mg/L, for ISV 4 mg/L–0.008 mg/L, for CLTZ 8 mg/L–0.0015 mg/L, and 4 mg/L–0.008 mg/L for AND. The prepared plates were stored at −75 °C until use. Before inoculation, the plates were defrosted at room temperature.

The cultured isolates were suspended in sterile distilled water to density of 0.5 McFarland. The suspensions were diluted tenfold in sterile distilled water and added to each well throughout the tested antifungal, ensuring an inoculum density of 0.5–2.5 × 10^5^ CFU/mL. The plates were later incubated at 35 °C for 24 h and absorbance readings were taken at 530 nm using a Multiskan Go microplate reader (Thermo Fisher Scientific, USA).

The minimum inhibitory concentration of the tested antifungal was determined as the lowest drug concentration at which the absorbance was reduced by 50% compared to the growth control of the test isolate.

#### 2.2.6. Concentration Gradient Strip Method

The concentration gradient strip technique was performed for anidulafungin and voriconazole [18]. After inoculating, the plates of RPMI 1640 supplemented with MOPS and 2% glucose medium plates (BioMaxima, Lublin, Poland), and the gradient test strips (Bio Merieux, Marcy-l’Étoile, France) were placed on the surface. Following 24–48 h incubation at 35 °C, a growth inhibition ellipse formed and the MIC values were identified at the point where the ellipse intersects the scale on the strip’s upper side.

The MIC results obtained with these three methods were presented using values described by parameters such as the minimum inhibitory concentration (MIC) range, MIC_50_ and MIC_90_ (the MIC values at which 50% or 90% of tested isolates were inhibited, respectively).

## 3. Results

**Figure 1:**
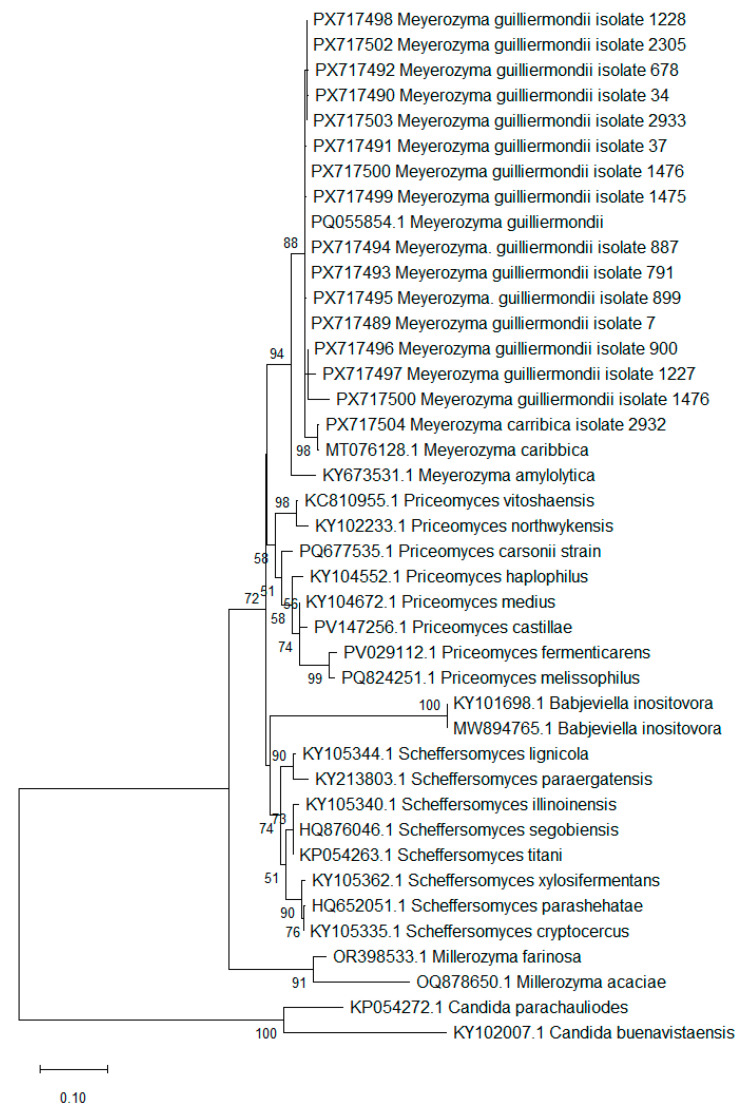
Phylogram representing the taxonomic placement of the studied isolates supporting molecular identification based on ITS sequences. Only nodes with frequency values higher than 50 are shown. Sequences with GenBank Accession Numbers PX17489-17504 were retrieved from the studied fungal isolates.

The MALDI-TOF method allowed for the identification of all but one of the tested isolates as *Meyerozyma guilliermondii sensu stricto*. The score for one isolate, No. 2932, was 1.7 for *Meyerozyma guilliermondii,* indicating probable identification at the genus level. Therefore, in order to ensure the accurate identification of this isolate, a molecular method was applied. (Supplementary Table S1; Figure 1).

The species identification of 16 out of the 20 studied isolates was confirmed through ITS rDNA region sequence analysis. For four of the studied isolates, ITS sequences could not be successfully obtained. The assembled sequences were used for downstream analysis. BLAST results (Table S1) identified 15 isolates as *Meyerozyma guillermondii* and a single isolate as *Meyerozyma caribbica*. This identification was further supported by a Maximum Likelihood phylogenetic analysis, which placed the studied strains with *Meyerozyma* spp. clade with high confidence. To corroborate the BLAST identification, a Maximum Likelihood phylogenetic reconstruction using the Tamura three-parameter model was conducted. The alignment included 41 ITS region nucleotide sequences representing 24 species across six genera—five genera belonging to the family of *Debaryomycetaceae*, including *Meyerozyma*, *Millerozyma*, *Priceomyces*, *Babjeviella* and *Scheffersomyces,* used to support taxonomic identification, and two *Candida* taxa used as an outgroup, based on the common order (*Saccharomycetales*) (Figure 1). The sequence matrix used in the analysis, after curation, consisted of 379 sites, including 185 variable sites and 150 parsimony-informative sites. The taxonomic placement of the studied isolates supported their molecular identification.

Drug susceptibility testing was performed for the entire population of tested isolates, which were classified as belonging to the *Meyerozyma guiellermondii* species complex (s.c.). 

Due to the fact that the results obtained for the anidulafungin using the first method were lower than expected according to EUCAST documents, two additional methods were used for this antifungal: the EUCAST reference microdilution method and the concentration gradient strip method using anidulafungin. To ensure the validity of the gradient strip method, voriconazole was also applied. All of the results are described below.

**Figure 2:**
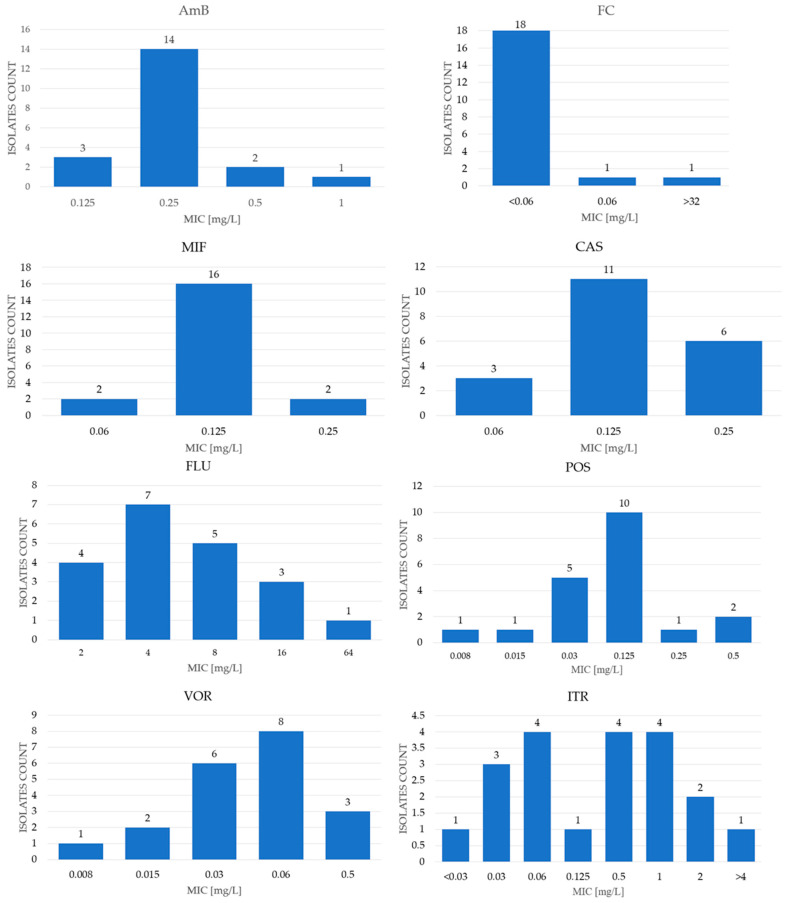
Distribution of MIC values of nine antimycotics for tested *Meyerozyma guilliermondii* s.c. isolates obtained using the MICRONAUT-AM test.

**Figure 3:**
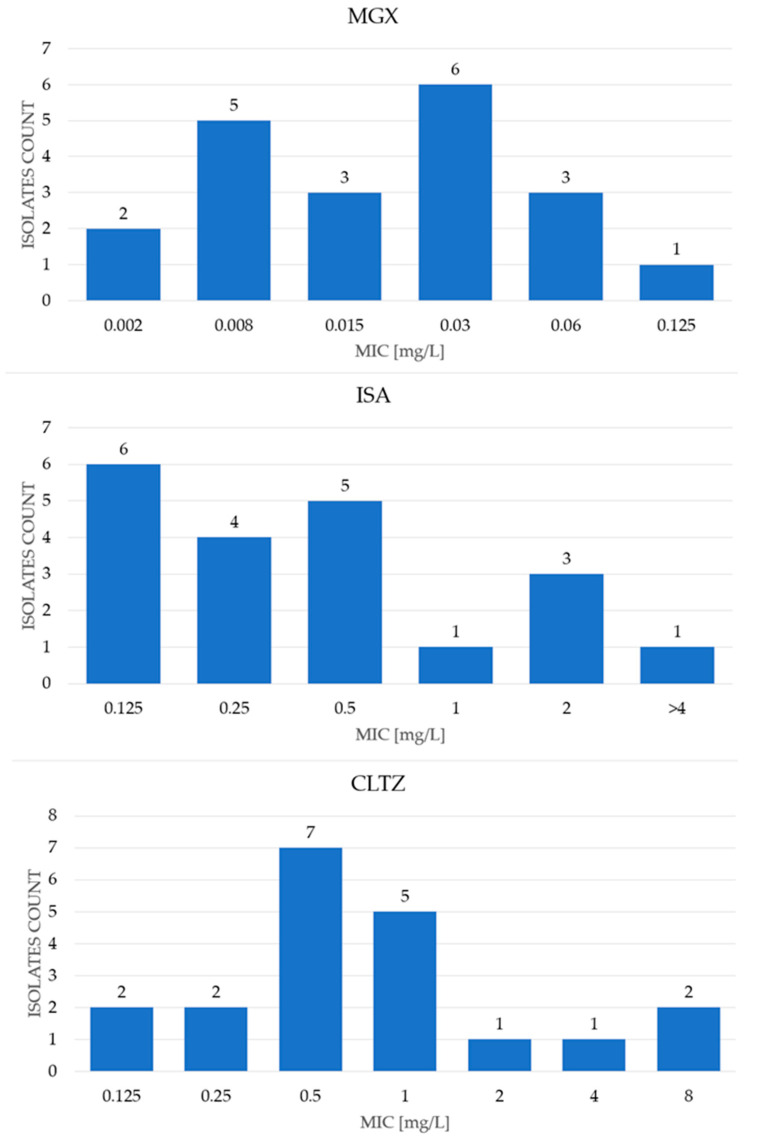
Distribution of MIC values of manogepix (MGX), isavuconazole (ISV) and clotrimazole (CLTZ) for tested *Meyerozyma guilliermondii* s.c. isolates obtained using the microdilution method according to EUCAST.

**Figure 4:**
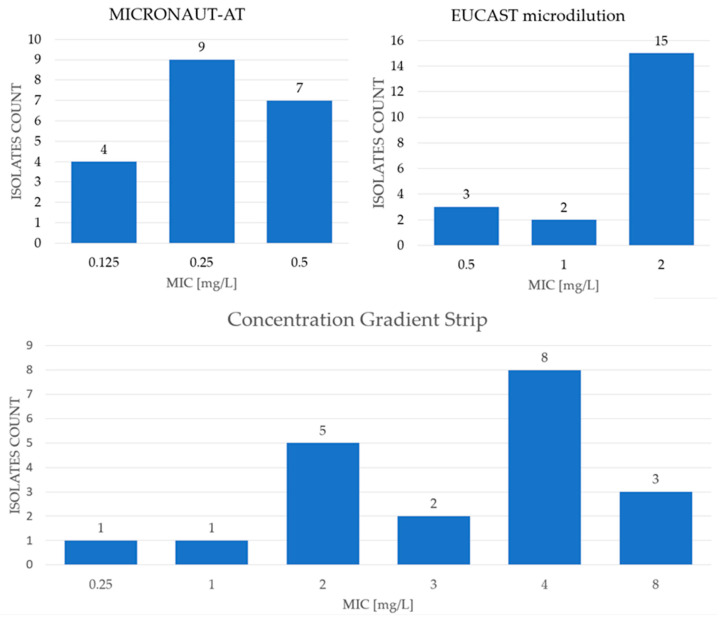
Distribution of MIC values of anidulafungin for tested *Meyerozyma guilliermondii* s.c. isolates obtained using three different methods.

The MIC ranges, as well as MIC_50_ and MIC_90_ values obtained for antifungals tested with the commercial MICRONAUT-AM test, are shown in Table 1 and in Figure 2, while the results obtained for antifungals tested with EUCAST microdilution method are presented in Table 1 and Figure 3. The results for anidulafungin determined by three different methods are presented in Table 1 and Figure 4.

The MIC range of amphotericin B for the tested isolates was 0.125–1 mg/L (Table S1, Table 1, and Figure 2). The MIC_50_ for AmB was 0.25 mg/L, and the MIC_90_ was 0.5 mg/L. Considering the tentative ECOFF value as 0.5 mg/L, 19 of the examined isolates were classified as WT (wild type), while one isolate was classified as NWT (non-wild type) [19].

As for flucytosine, EUCAST does not provide susceptibility data. The MIC values for the tested isolates were, however, very low, with an MIC_90_ of 0.06. Only one blood isolate showed a MIC value of >32 mg/L, which may suggest resistance.

The MIC range for fluconazole was 2–64 mg/L, with a MIC_50_ and MIC_90_ of 4 mg/L and 16 mg/L, respectively. According to EUCAST guidelines, the ECOFF value for *Meyerozyma guiellermondii* is established at 16 mg/L [19]; therefore, one strain was classified as NWT. For voriconazole (VOR) and posaconazole (POS), the MIC ranges were identical: 0.008–0.5 mg/L. The MIC_50_ for both antifungals was 0.0625 mg/L, while the MIC_90_ values were 0.125 mg/L for VOR and 0.25 mg/L for POS. Since EUCAST provides an ECOFF value of 0.25 mg/L only for POS, two isolates were categorized as NWT [12]. However, according to the guide for rare species without breakpoints, for isolates with a MIC value for VOR of ≤0.125 mg/L, VOR may be considered for treatment. So, two of the isolates under study were out of this group. The concentration gradient strip method was also applied for VOR, yielding a MIC range of 0.012–0.19 mg/L. The results obtained with both methods were generally comparable for each strain, with only minor differences related to the visual MIC interpretation (Table S1 and Table 1).

The MIC range for itraconazole was from ≤0.03 mg/L to ≥4 mg/L, with MIC_50_ and MIC_90_ values of 0.5 mg/L and 1 mg/L, respectively. The ECOFF value was established at 1 mg/L; therefore, 17 tested isolates were classified as WT and three isolates as NWT [19]. For the newer azole, isavuconazole, the MIC range was also wide, from 0.06 mg/L to >4 mg/L, with a MIC_50_ of 0.25 mg/L and MIC_90_ of 2 mg/L. For clotrimazole, the MIC range was 0.06 mg/L to >8 mg/L, with MIC_50_ and MIC_90_ values of 0.5 mg/L and 2 mg/L, respectively. However, in these cases, the ECOFF value was not established; therefore, the EUCAST-based classification was not possible.

For manogepix, the MIC range was 0.002 mg/L–0.125 mg/L, with MIC_50_ and MIC_90_ values of 0.015 mg/L and 0.06 mg/L, respectively (Table S1 and Table 1, Figure 3) [20]. As this is a novel drug, EUCAST has not yet defined an ECOFF value.

For echinocandines tested using the MICRONAUT-AM method, the obtained MIC values were rather low (Table S1 and Table 1, Figure 2). The MIC range for anidulafungin (AND) was 0.125 mg/L–0.5 mg/L with MIC_50_ and MIC_90_ values of 0.25 mg/L and 0.5 mg/L, respectively. The MIC ranges for micafungin (MIF) and caspofungin (CAS) were identical: ≤0.06 mg/L–0.25 mg/L. The MIC_50_ values were the same for both antifungals: 0.125 mg/L, while MIC_90_ values were 0.125 mg/L for MIF and 0.25 mg/L for CAS (Table 1; Figure 2).

However, according to the latest EUCAST update [19], the ECOFF value for anidulafungin is as high as 2 mg/L. Based on these guidelines, all the tested isolates should be considered as WT. Therefore, the authors decided to verify the obtained results using the EUCAST reference microdilution method and the concentration gradient strip method. Using these methods, the MIC range for anidulafungin was higher: 0.5–2 mg/L, with a MIC_50_ and MIC_90_ value of 2 mg/L. Applying different testing methods, the obtained results suggest that all the isolates should be still classified as WT. However, it should be noted that the obtained results differed according to the method used. The concentration gradient strip method was also applied for this antifungal and the MIC value was even higher: 0.25–8 mg/L, with MIC_50_ and MIC_90_ values of 4 and 8 mg/L, respectively (Table 1, Figure 4).

Regarding EUCAST, quality control isolates *Candida krusei* ATCC 6258 and *Candida parapsilosis* ATCC 22019 were used. All the gained results were consistent with the MIC range established by EUCAST for this reference isolates (Table S1).

## 4. Discussion

The deep-seated infections caused by *Meyerozyma guilliermondii* pose serious challenges in the diagnosis and therapy, especially for immunosuppressed patients. For example, fungemia, due to *Meyerozyma guilliermondii*, is characterized by a high mortality rate up to 58% and the optimal treatment has not been established [8,21]. These infections are relatively rare in our region, but when they do occur, they pose a serious therapeutic problem. In this study we tested an older collection of clinical isolates, originally identified on the basis of their metabolic characteristics, and our first task was to re-identify them using the currently available MALDI-TOF mass spectrometry method. A limitation of this approach is that MALDI-TOF cannot differentiate the members of the *Meyerozyma guilliermondii* species complex. Therefore, to determine whether cryptic species such as *Meyerozyma caribbica* (*Candida fermentati*) and *Candida carpophila* were present among tested *M. guilliermondii* clinical isolates, the authors decided to perform the molecular identification method [22]. Based on ITS sequence analysis, the authors were able to identify one *Meyerozyma caribbica* isolate among the studied clinical isolates, which was the only isolate that MALDI-TOF failed to classify to the species level. According to our knowledge, this is the first report of *Meyerozyma. caribbica* identification in our region; however, according to global data this species may be responsible for up 16% of fungemia cases attributed to *Meyerozyma guilliermondii* s.c. [9].

A limitation for the routine drug susceptibility testing of yeasts has long been the lack of appropriate commercial tests. The reference methods, considered the gold standard, are highly labour intensive and therefore are rarely used in routine practice. Currently, commercial panels based on CLSI or EUCAST principles have been available on the market for several years, including MICRONAUT-AM. The results obtained using different methods may vary; however, MICRONAUT-AM shows high concordance with the reference method, and, according to the manufacturer’s information, it can be interpreted in accordance with the EUCAST criteria. Therefore, the authors decided to use this system in the present study. Additionally, to explore new potential therapeutic options, the study was extended to include additional antifungal agents, such as manogepix, isavuconazole and clotrimazole, which were tested using the EUCAST microdilution method [17].

One of the major challenges in the susceptibility testing of rare species is not the performance of the test itself, but the interpretation of the obtained results. Due to the limited knowledge and rarity of these microorganisms, clinical breakpoints are lacking and ECOFF values have been attained for only a few antifungal agents. A valuable contribution in this area was provided by the most recent EUCAST update [19] and by a study published in October 2024 by Jack W. McHugh et al. [8], in which 112 of *Meyerozyma* spp. isolates were tested. Nevertheless, the authors sought to compare the results of present study with the available literature data (Table 2). Several difficulties were encountered, mainly because some authors used different criteria or did not use the ECOFF value for classification. The available medical literature contains only a few studies on the drug susceptibility of *Meyerozyma guilliermondii.* As mentioned previously, no comprehensive study with regard to *Meyerozyma guilliermondii* has been conducted in Poland.

The MIC values obtained for amphotericin B in the MICRONAUT test were consistent with those reported in the other studies [8,23,24]. Overall, the susceptibility to this antifungal remains high, with only few isolates classified as NWT in the cited studies. EUCAST provided a tentative ECOFF value 0.5 mg/L for amphotericin B (AmB); however, the recommendation for rare yeast without breakpoints suggests that isolates with the MIC values of ≤1 mg/L may still be considered for therapy. Although resistance to polyenes in the *M. guilliermondii* species complex is rarely reported, some studies indicate the possibility of reduced susceptibility. For example, in a study conducted in Slovakia, out of 262 fungal isolates from bloodstream infections, one of the seven *M. guilliermondii* isolates showed potential resistance to nystatin and amphotericin B (MIC 4 mg/L). Similarly, a study conducted in India in 2015, involving 70 HIV-seronegative and -seropositive patients, reported one *M. guilliermondii* NWT strain, with an MIC value of ≥4 mg/L [25].

Currently, EUCAST does not provide breakpoints for flucytosine for any yeast-like fungi species. The results obtained in the present study are consistent with those reported by McHugh et al. [8], with a single isolate exhibiting a very high MIC value (32 and 64 mg/L respectively). In a study by Tseng et al. [24], a value of 1.0 mg/L was adopted as the epidemiologic cut-off value. Applying this criterion, 21 clinical isolates from the present study (representing 95.5% of the study population) would be classified as WT, while one isolate as NWT (MIC 32 mg/L). This is consistent with the results obtained by Cuenca-Estrella et al. [26], where 55% of the population was classified as susceptible and 45% as potentially susceptible (with MIC range of <0.25–16 mg/L).

Regarding the azoles, one isolate exhibited elevated MIC values for all the tested azoles (no. 34; Table S1), while several others were classified as NWT for one or two azoles. Resistance to azoles may develop through multiple mechanisms, including mutations in *ERG* genes, efflux pump overexpression and other regulatory changes, often resulting in cross-resistance within the azole class. The recommendation for interpreting MIC for rare yeasts without clinical breakpoints according to EUCAST suggests adopting a value of 16 mg/L for FLU, 0.25 mg/L for POS ≤ 0.125, and 1 mg/L for ITR [19]. For voriconazole, 90% of isolates did not exceed a MIC value of 0.125 mg/L, which, according to EUCAST, suggests that the drug may be considered for use in certain clinical situation [27]. In a study conducted by Sriphannam et al. [28], the MIC breakpoint of >8 mg/L was applied for *M. guilliermondii*, resulting in approximately 67% of the study isolates being considered as resistant (MIC range 3.0–>256.0 mg/L). Among the isolates tested using the MICRONAUT-AM method, the percentage of isolates potentially resistant to fluconazole was relatively low, at 20% only, using a cut-off point of >8 mg/L. This aligns with the results reported by Tseng et al. [24], who classified 31.8% of isolates as resistant at a cut-off value of >8 mg/L. Large-scale analysis from the ARTEMIS DISK Antifungal Monitoring Programme of 1029 clinical isolates of *M. guilliermondii*, collected from 127 medical facilities, demonstrated the limited susceptibility of *M. guilliermondii* to fluconazole. [29]. Only 75% of the isolates were susceptible to this drug, while voriconazole showed higher efficacy in vitro. Differences in drug activity depending on the type of isolates are also significant. Isolates from dermatology or surgery showed a lower susceptibility to fluconazole compared to blood-derived isolates. Studies on the susceptibility of *Meyerozyma guilliermondii* to fluconazole and voriconazole have shown significant geographical variation, which is crucial for therapeutic strategies and the management of fungal infections. It should be noted that although *M. guilliermondii* shows limited susceptibility to fluconazole, it remains sensitive to voriconazole at commonly used clinical doses. Nevertheless, variability in susceptibility to these drugs is observed depending on the geographical region and type of clinical sample. Blood isolates often show a similar drug susceptibility profile, while isolates from skin and soft tissue infections may show reduced susceptibility to fluconazole [29].

For posaconazole (POS), 18 isolates were classified as wild type based on the ECOFF value. Comparing to other studies, lower values were obtained for this antifungal agent [8,24]. The inhibitory concentration for itraconazole (ITR) ranged widely, from <0.03 to >4 mg/L, and 17 isolates were designated as wild type (WT). One isolate (No. 1475) was classified as NWT for both POS and ITR. Da Matta et al. [30] adopted a lower MIC cut-off value for ITR of ≤0.125 mg/L, resulting in two isolates being classified as NWT. Applying the same criterion to the present study would indicate that 55% of the tested isolates could be considered potentially resistant to itraconazole.

Isavuconazole is primarily indicated for the treatment of aspergillosis and mucormycosis, but recent studies have explored its activity against uncommon yeast species [31,32]. No ECOFF value has yet been established for this drug. Nevertheless, three of tested isolates exhibited an elevated MIC value (2 mg/L). Desnos-Ollivier et al. tested 23 *M. guillermondii* isolates (21 were cultured from blood samples) for isavuconazole susceptibility. The results were similar to the obtained results in this study [31]. However, the results of the study performed by Badiee et al. indicated lower MIC values for the tested population (12 isolates) with an MIC range of 0.008–0.125 mg/L [32]. Moreover, the authors also tested other antifungals, which are shown in Table 2. Another study also performed in Poland included two *M. guilliermondii* isolates, which also exhibited lower MIC values (0.002 mg/L and 0.0064 mg/L) [10]. Therefore, the knowledge gap concerning this antifungal is still huge and this area needs more investigation.

As far as clotrimazole (CLZ) is concerned, it is hard to find research with *Meyerozyma guilliermondii.* However, since it can be found on skin and, therefore, can cause some infections, expanding research in this area should be considered. In a previous study by our team, the CLZ susceptibility of 125 genitourinary isolates of *Candida* spp. were tested and the obtained MIC ranged from 0.008 to 8 mg/L, and the highest MIC (8 mg/L) was observed in the FLU-resistant isolates of *Candida glabrata* and *Candida albicans*. A similar pattern was observed among *Meyerozyma guilliermondii*, where a MIC of 8 mg/L displayed only one strain identified as FLU resistant [33].

An additional challenge was encountered with anidulafungin MIC values. As described previously, the susceptibility testing was repeated using the EUCAST reference microdilution method. Not only did the obtained MIC values not match in both methods, but they also turned out to be higher, nearly matching the ECOFF value suggested by EUCAST. Therefore, another method was introduced to the research—the method with gradient concentration strips. The results matched or differed by a maximum of two 2-fold dilutions for each strain. It should be emphasized here that the voriconazole gradient tests used for comparison showed almost complete agreement with the MICRONAUT method (Table S1 and Table 1). Based on that it is important to carefully interpret the MICRONAUT results concerning echinocandins. The commercial test was chosen in order to reflect the working conditions in a routine laboratory, allowing the authors to receive the susceptibility profile to common antifungal drugs. The commercial test MICRONAUT-AM is based on the EUCAST method, and although the difference lies in the reading of the results, most authors show good, though not 100%, consistency. The commercial panels have many advantages in routine laboratory practice: they are simpler to use and allow for the testing of multiple drugs at once, and the tools and solutions are ready-made. What is more, in both methods, the results for the reference strain were identical and consistent with EUCAST [34].

*Meyerozyma guilliermondii* s.c. is characterized by the naturally elevated MIC of echinocandins resulting from Fks1 gene polymorphism. In the isolates population examined in the present study, the MIC of anidulafungin was rather low with MICRONAUT-AM tests (MIC_50_ of 0.25 mg/L and MIC_90_ of 0.5 mg/L) but 2–4 times or even 16 times higher when EUCAST or gradient strip tests were used (Figure 4). In the case of echinocandins, there is a high diversity in the clinical breakpoint values (CBPs) for different yeast species. For example, the CBP of anidulafungin for *Candida parapsilosis* is 4 mg/L, while for *C. glabrata* and *C. krusei*, it is 0.06 mg/L [19]. For the isolates of *M. guilliermondii*, Tseng et al. [24] suggest adopting a CBP value of >2 mg/L. Using the above criteria, all isolates should be classified as WT in MICRONAUT-AM as well as EUCAST methods, which correlates with the results published by the researchers, where 72.7% of isolates showed susceptibility to anidulafungin. The results obtained with gradient strip tests, although differing in 1–2 dilutions from EUCAST, show strong categorical discrepancy due to the small pool of strains. No conclusions can be drawn about correlation, but they should not be ignored either. While differences between the MICRONAUT-AM/EUCAST reference method do not change the categorization, the gradient strip and EUCAST method do (20 WT versus 13/20 non-WT). In terms of the MIC values for caspofungin, the value range between <0.06 and 0.25 mg/L was observed, which is lower than that of the results presented in the publication by Tseng et al. [24], where the value range was 0.25 to 8 mg/L. In the case of caspofungin, due to technical problems, no CBP or ECOFF was established by EUCAST. However, it can be assumed that isolates susceptible to anidulafungin and micafungin can also be considered susceptible to caspofungin. For micafungin, the MIC range was <0.06 to 0.25 mg/L, which is significantly lower than that obtained in the study by Tseng et al. [24], where the range was 0.25 to 8 mg/L. If we assume that the CBP value does not exceed 2 mg/L, all isolates tested by MICRONAUT-AM should be classified as susceptible, which confirms the consistency with the results obtained by researchers who obtained 90.9% of potentially susceptible isolates [24].

In extensive studies published by Arendrup et al. in 2020, which covered many yeast species, nine isolates of *Meyerozyma guilliermondii* were tested for sensitivity to manogepix [23]. The researchers reported MIC values ranging from 0.002 to 0.06 mg/L, which is consistent with the results obtained in this study. The MIC values reported ranged from 0.002 to 0.125 mg/L. In addition, they determined MIC_50_ and MIC_90_ values of 0.004 mg/L and 0.06 mg/L, respectively. MIC_50_ slightly differs from the value obtained in the study (0.015 mg/L), while MIC_90_ is consistent (0.06 mg/L) [23]. However, the tested population in both cases is still low, what clearly shows the knowledge gap in this area.

The resistance of *Meyerozyma guilliermondii* to drugs is a phenomenon that may pose a serious clinical problem. The observed variability of resistance of *M. guilliermondii* to antifungal agents highlights the importance of the systematic monitoring of antifungal resistance and the necessity of adjusting therapy according to susceptibility testing results. Therefore, it is important to develop new treatment strategies that take into account the diverse drug susceptibility profiles of different yeasts populations, including the use of the therapeutic potential of substances such as manogepix [29].

The obtained data confirm that the susceptibility testing and the interpretation of the results for *Meyerozyma guilliermondii* may pose significant problems, especially regarding echinocandins (with the commercial methods) as well as in relation to FC, for which there are no ECOFF or clinical breakpoints. According to the obtained results, AMB retains good activity and MGX seems to be a good candidate for treatment based on in vitro studies.

**Table 2:** *Meyerozyma guilliermondii* antifungal susceptibility results obtained in personal research (PR) and data published by other authors.

Antifungal	Source	n	MIC Range [mg/L]	MIC_50_	MIC_90_	ECOFF [mg/L]	WT (%)	NWT (%)
AmB	PR^M^	20	0.125–1	0.25	0.5	0.5	95	1
	[8]	112	0.125–1	0.5	1		88.4	11.6
	[23]	9	0.125–1	0.25	1		88.88	11.11
	[24]	22	0.25–8	0.5	0.5		95.5	4.5
	[32]	12	0.016–0.5	0.125	0.5		-	-
FC	PR^M^	20	<0.06–32	0.06	0.06	8	95	5
	[8]	112	<0.06–64	0.06	0.12	-	-	-
	[24]	22	<0.06–2	≤0.06	0.25	1	95.5	4.5
	[26]	20	≤0.25–16	2	0.25	8	55	45
FLU	PR^M^	20	2–64	4	16	16	95	5
						8	80	20
	[8]	112	0.125–>256	4	32	16	85.7	14.3
	[23]	9	2–>64	8	>64	-	-	-
	[24]	22	0.5–256	8	64	8	68.2	31.8
	[26]	20	0.25–64	8	64	-	-	-
	[28]	3	3.0–>256	-	-	8	33	67
	[32]	12	0.25–16	1	16	-	-	-
VOR	PR^M^	20	0.008–0.5	0.06	0.125	-	-	-
	[8]	112	≤0.008–8	0.06	2	-	-	-
	[24]	22	0.015–8	0.12	0.1	0.25	63.6	36.4
	[28]	9	0.03–4	0.125	4	-	-	-
	[32]	12	0.016–0.125	0.032	0.125			
POS	PR^M^	20	0.008–0.5	0.06	0.25	0.25	90	10
	[8]	112	0.015–2	0.25	0.5	0.25	68.7	31.3
	[24]	22	0.03–8	0.5	1	0.5	72.7	27.2
	[32]	12	0.016–8	0.5	8	-	-	-
ITR	PR^M^	20	≤0.03–≥4	0.5	1	1	85	15
	[8]	112	0.03–16	0.25	1	1	90.2	9.8
	[24]	22	0.03–8	0.5	4	0.25	-	-
	[26]	20	0.03–2	0.5	0.5	-	-	-
	[30]	30	-	0.25	0.25	0.125	43.4	6.6
	[32]	12	0.016–8	0.25	0.5	-	-	-
MIF	PR^M^	20	≤0.06–0.25	0.125	0.125	2	100	0
	[8]	112	0.06–2	0.5	1	-	-	-
	[24]	22	0.25–8	1	2	2	90.9	4.5
	[28]	9	0.125–0.5	0.25	0.5	-	-	-
AND	PR^M^	20	0.125–0.5	0.25	0.5	2	100	0
	[8]	112	0.125–2	1	2	-	-	-
	[24]	22	0.5–8	2	4	2	72.7	9.1
	[28]	9	0.25–1	0.5	1	-	-	-
CAS	PR^M^	20	≤0.06–0.25	0.125	0.25	-	-	-
	[8]	112	0.06–8	0.25	0.5	-	-	-
	[24]	22	0.25–8	0.5	>8	2	77.3	22.7
	[29]	132	0.03–>8	0.5	1	-	-	-
	[32]	12	0.016–1	0.25	4	-	-	-
ISA	PR^E^	20	0.06–2	0.25	2	-	-	-
	[10]	2	0.002–0.0064	-	-	-	-	-
	[31]	23	0.03–>4	0.25	2	-	-	-
	[32]	12	0.008–0.125	0.064	0.125	-	-	-
MGX	PR^E^	20	0.002–0.125	0.015	0.06	-	-	-
	[23]	9	0.002–0.06	0.004	0.06	-	-	-

PR^M^—personal research; MICRONAUT-AM method. PR^E^—personal research; EUCAST method. n—number of tested isolates. Not determined, because of lack of ECOFF.

## Figures and Tables

**Table 1:** The summary of MIC ranges, MIC_50_ and MIC_90_ values of twelve investigated antifungals obtained for *M. guilliermondii* s.c. isolates, according to the applied method.

Method	Antifungal	MIC Range [mg/L]	MIC_50_	MIC_90_
MICRONAUT	AmB	0.125–1	0.25	0.5
	FC	≤0.06–≥32	0.06	0.06
	FLU	2–64	4	16
	ITR	≤0.03–≥ 4	0.5	1
	VOR	0.008–0.5	0.06	0.125
	POS	0.008–0.5	0.06	0.25
	MIF	≤0.06–0.25	0.125	0.125
	AND	0.125–0.5	0.25	0.5
	CAS	≤0.06–0.25	0.125	0.25
EUCAST [17]	ISA	0.06–2	0.25	2
	CLTZ	0.125–8	0.5	4
	MGX	0.002–0.125	0.015	0.06
	AND	0.5–2	2	2
Concentration Gradient Strip	AND	0.25–8	4	8
	VOR	0.012–0.19	0.032	0.064

Abbreviations: MIC_50_—the MIC values at which 50% tested isolates were inhibited; MIC_90_—the MIC values at which 90% of tested isolates were inhibited; AmB—amphotericin B; FC—flucytosine; FLU—fluconazole; VOR—voriconazole; POS—posaconazole; ITR—itraconazole; AND—anidulafungin; MIF—micafungin; CAS—caspofungin; MGX—manogepix; ISA—isavuconazole; and CLTZ—clotrimazole.

## Data Availability

The original data is presented in the article and in the supplement (Table S1). For more information, please contact the corresponding author.

## Supplementary Materials

The following supporting information can be downloaded at https://www.mdpi.com/article/10.3390/pathogens15020235/s1.
